# Dynamic Vibrotactile Signals for Forward Collision Avoidance Warning Systems

**DOI:** 10.1177/0018720814542651

**Published:** 2015-03

**Authors:** Fanxing Meng, Rob Gray, Cristy Ho, Mujthaba Ahtamad, Charles Spence

**Affiliations:** Tsinghua University, Beijing, China; University of Birmingham, Birmingham, United Kingdom; University of Oxford, Oxford, United Kingdom; University of Birmingham, Birmingham, United Kingdom; University of Oxford, Oxford, United Kingdom

**Keywords:** driving, haptic, interface design, front-to-rear-end collision, car following, break reaction time

## Abstract

**Objective:**

Four experiments were conducted in order to assess the effectiveness of dynamic vibrotactile collision-warning signals in potentially enhancing safe driving.

**Background:**

Auditory neuroscience research has demonstrated that auditory signals that move toward a person are more salient than those that move away. If this looming effect were found to extend to the tactile modality, then it could be utilized in the context of in-car warning signal design.

**Method:**

The effectiveness of various vibrotactile warning signals was assessed using a simulated car-following task. The vibrotactile warning signals consisted of dynamic toward-/away-from-torso cues (Experiment 1), dynamic versus static vibrotactile cues (Experiment 2), looming-intensity- and constant-intensity-toward-torso cues (Experiment 3), and static cues presented on the hands or on the waist, having either a low or high vibration intensity (Experiment 4).

**Results:**

Braking reaction times (BRTs) were significantly faster for toward-torso as compared to away-from-torso cues (Experiments 1 and 2) and static cues (Experiment 2). This difference could not have been attributed to differential responses to signals delivered to different body parts (i.e., the waist vs. hands; Experiment 4). Embedding a looming-intensity signal into the toward-torso signal did not result in any additional BRT benefits (Experiment 3).

**Conclusion:**

Dynamic vibrotactile cues that feel as though they are approaching the torso can be used to communicate information concerning external events, resulting in a significantly faster reaction time to potential collisions.

**Application:**

Dynamic vibrotactile warning signals that move toward the body offer great potential for the design of future in-car collision-warning system.

## Introduction

In the past few years, there has been a great deal of interest in the development of assistance systems, in particular, nonvisual and multisensory collision-warning systems for drivers (e.g., Fitch, Kiefer, Hankey, & Kleiner, 2007; Ho, Reed, & Spence, 2006; Lee, McGehee, Brown, & Marshall, 2006; Mohebbi, Gray, & Tan, 2009; Spence, 2012; Spence & Ho, 2008a, 2008b). It has been suggested that such systems present a solution that may potentially contribute significantly to reducing the number and severity of rear-end collisions on our roads (see Kiefer et al., 1999; Tijerina, Johnston, Parmer, Pham, & Winterbottom, 2000; Young, Lee, & Regan, 2008). Research in the area of collision-warning signal design has demonstrated the potential benefits that may be associated with the presentation of auditory, tactile, and multisensory warning signals in alerting a driver and rapidly orienting his or her spatial attention in the direction of the potential danger (for reviews, see Haas & van Erp, 2014; Spence & Ho, 2008a; see also Baldwin et al., 2012; Deatherage, 1972; Gray, 2011; Ho & Spence, 2008; Ho, Spence, & Tan, 2005; Lee, Hoffman, & Hayes, 2004; Lee, McGehee, Brown, & Reyes, 2002).

In a realistic driving environment, however, the effectiveness of auditory warning signals may be limited due to the fact that some drivers suffer from a hearing impairment (McKeown & Isherwood, 2007). In addition, some auditory warning signals may be easily confused with background noise under everyday driving conditions (see Beruscha, Augsburg, & Manstetten, 2011; COMSIS, 1996; McKeown & Isherwood, 2007; Ramsey & Simmons, 1993). Alternatively, they may perhaps be interfered with by other auditory tasks, such as talking to a passenger or using a mobile phone (see Mohebbi et al., 2009).

By contrast, the sense of touch is generally far less central to driving than either vision or audition, although it plays a role in the perception of vehicle acceleration and vibration (Hogema, De Vries, van Erp, & Kiefer, 2009). Therefore, the sense of touch could potentially provide a readily available channel to present warnings to the driver. It has been noted previously that some practical issues, such as the possibility that certain drivers might be insensitive to tactile stimuli (see Thornbury & Mistretta, 1981), or that there may be some sort of masking effect elicited by the whole-body vibration experienced by the driver on the road (see Ryu, Chun, Park, Choi, & Han, 2010), not to mention any insensitivity that could result from drivers wearing thick clothing/gloves on their ability to perceive the tactile cues (see Spence & Ho, 2008b), might be expected to degrade the effectiveness of vibrotactile warnings.

As compared with other types of warning signals, it has been suggested that tactile warnings are highly intuitive and may be associated with rapid responses (e.g., Prewett, Elliott, Walvoord, & Coovert, 2012). Previous research has revealed that tactile warning signals can sometimes be more effective than the equivalent auditory or visual warning signals (for a review, see Prewett et al., 2012; see also Deatherage, 1972; Fitch et al., 2007; Ho & Spence, 2008; Spence & Ho, 2008b). For example, the results of a study by Mohebbi et al. (2009) demonstrated that simple, casual cell phone conversations may render auditory warnings ineffective (i.e., no significant reduction in braking reaction times [BRTs] were observed when performance was compared to a no-warning condition). However, the conversation did not affect the effectiveness of the tactile warnings. Previous research has also demonstrated that people can respond more rapidly to vibrotactile warning signals than to either auditory or visual warnings in potential road collision events (see Ho, Spence, & Tan, 2005; Scott & Gray, 2008; Terrence, Brill, & Gilson, 2005). Taken together, then, many studies have demonstrated an improvement in the behavioral performance of drivers to time-critical events resulting from the use of vibrotactile warning signals (Ho, Spence, & Tan, 2005; Murata, Tanaka, & Moriwaka, 2011; Sklar & Sarter, 1999; Spence & Ho, 2008b).

Related research also suggests that stimuli presented in a driver’s peripersonal space (i.e., those stimuli that are presented around a driver’s body, cf. Previc, 1998, 2000) might offer a substantive advantage in terms of alerting a driver to potential collision events (see Ho & Spence, 2009). However, it is still a challenge to convey information about those potential collision events currently situated in extrapersonal space (i.e., located at a greater distance from the driver than occupied by peripersonal space, that is, the space he or she can reach) via tactile warning signals (which are usually presented in personal space).

In the case of auditory warnings, Gray (2011) has designed an auditory looming warning, whose intensity increased as the distance between the driven vehicle and the lead vehicle decreased, to convey information concerning the urgency of the potential collisions with the lead vehicle to the driver. Comparison between auditory looming warning and other, nonlooming warning signals has demonstrated that the auditory looming signal can lead to significantly speeded BRTs. The effectiveness of auditory looming signals may be explained by the natural mapping between the auditory signals and the external events that they reference (see Petocz, Keller, & Stevens, 2008): An auditory signal whose intensity increases provides an impression that the sound-emitting object is approaching the driver (see Shaw, McGowan, & Turvey, 1991), which is more salient than the nonlooming signal.

It is therefore interesting to investigate whether the mechanism underlying the detection of looming auditory warnings also extends to the tactile modality. The aim of the present study was to examine whether a looming tactile warning can also convey information concerning the danger approaching the driver, thereby potentially speeding his or her braking response to an impending collision. However, the majority of the vibrotactile warning signals that have been trialed previously were static (that is, no physical variation in the tactile signals was involved; see Chun, Han, Park, Seo, & Choi, 2012; Mohebbi et al., 2009). Thus, few researchers have directly investigated looming effects in tactile perception. One of the only examples of research that has focused on the consequence of presenting such looming vibrotactile warnings was reported by Ho, Spence, and Gray (2013). The participants in this study were presented with a single tactor whose vibration intensity increased rapidly over a short space of time. This stimulus constituted a looming vibrotactile cue. However, the results indicated that the looming vibrotactile warnings did not stand out from the other, nonlooming vibrotactile warnings that were tested in the same study, either in terms of their ability to facilitate participants’ BRTs or in terms of their ability to reduce the incidence of false-alarm braking.

Ho et al.’s (2013) results demonstrate that the design of looming vibrotactile warning signals is not simply a matter of making a pattern of vibrotactile stimulation that matches that seen in the auditory modality. In particular, the presentation of looming-intensity information at a single body location appears to be ineffective in terms of conveying approach information that is capable of preparing a driver’s response to an impending collision. Furthermore, the effectiveness of this type of warning is not improved when the rate of increase of vibrotactile intensity is linked to closing velocity (Experiment 1 in Gray, Ho, & Spence, 2014). Given that the tactile stimulus is perceived as being close to (or in contact with) the driver’s skin, it would appear that increasing intensity alone cannot provide a natural mapping to an approaching external danger.

Currently, dynamic vibrotactile warnings (i.e., introducing some dynamic qualities into the vibrotactile stimuli) are seldom studied in the context of in-vehicle collision warnings. Related studies have suggested that the urgency of a warning signal can be enhanced by adding more dynamic qualities (Edworthy & Hellier, 2006; Haas & Edworthy, 2006). Therefore, the present study was designed to further investigate whether the dynamic vibrotactile warnings, which involved vibrotactile stimuli that traveled from the driver’s hands toward the driver’s torso, would convey approach information concerning an impending forward collision to the driver, thus facilitating his or her braking responses.

Usually, when a driver assumes a typical driving posture, his or her arms are extended out in front of him or her while holding onto the steering wheel. In Experiment 1 of the present study, the effectiveness of vibrotactile warning signals that traveled from the participant’s hands toward his or her torso (waist) was compared to those that traveled away from the torso toward the hands. Given that the toward-torso vibrotactile warnings matched the direction of a potential forward collision event, we hypothesized that the presentation of vibrotactile signals traveling toward the driver’s torso might facilitate speeded braking responses, as compared to vibrotactile cues that appeared to be moving away from his or her torso.

## Experiment 1

### Participants

Sixteen participants between the ages of 21 and 40 (mean age of 28 years) took part in this experiment. All of the participants held a full driving license, and had been driving for between 1 and 23 years (mean of 10 years). Seven of the participants reported that they would normally drive on the left side of the road (i.e., as is normally the case in England and many Commonwealth countries), and the remainder reported that they would normally drive on the right. All of the participants reported having normal color vision, normal or corrected-to-normal vision, normal hearing, a normal sense of touch, and no history of neck pain. The experiment lasted for about 30 min, and each participant completed an informed-consent form and received £5 in return for their participation. The experiment was conducted in accordance with the ethical guidelines laid down by the Central University Research Ethics Committee, University of Oxford.

### Apparatus and Materials

**Figure 1.**
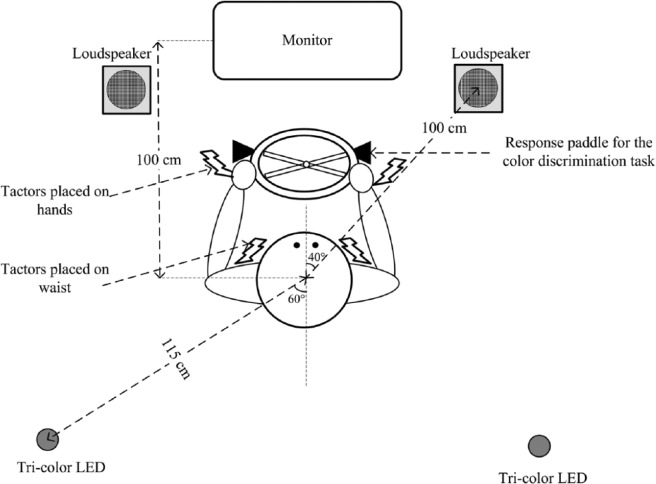
Schematic bird’s-eye view of the setup used in the laboratory-based Experiments 1, 3, and 4.

The same setup described in Ho, Gray, and Spence (2014) was used in this experiment. In particular, the participants were seated in front of a steering wheel (Logitech MOMO Racing Force Feedback Wheel; Logitech, Inc., Fremont, CA, USA) attached to a desk in a completely dark experimental booth (220 cm × 200 cm). The accelerator and brake pedals were placed on the floor at a comfortable distance from their right foot. Two hand paddles mounted behind the steering wheel were used to collect the participants’ responses to a color discrimination task described in detail later (see Figure 1). A computer monitor (51.0 cm × 28.3 cm), with a refresh rate of 60 Hz, was placed at a viewing distance of 100 cm directly in front of the participants in order to present the frontal simulated driving stimuli. A red-green-blue tricolor light-emitting diode (LED) was placed about 115 cm behind the participants’ back and approximately 60° to the left and right of the participants’ body midline (at an elevation that roughly matched their shoulder level when seated) in order to present the visual targets for the color discrimination task.

Vibrotactile stimuli were presented via four tactors (VBW32, Audiological Engineering Corp., Somerville, MA, USA). One tactor was fastened to the back of each of the participant’s hands and the other to the left and right sides of the participant’s waist using Velcro belts. The tactors were driven by a 250 Hz sinusoidal signal at an intensity that was sufficient to deliver clearly perceptible vibrotactile stimuli. Two types of vibrotactile stimuli were presented: toward-torso and away-from-torso cues. The toward-torso cue consisted of the simultaneous operation of both tactors on the participant’s hands for 150 ms, immediately followed by the simultaneous operation of both tactors around their waist for 150 ms (i.e., the interstimulus interval was 0 ms). The away-from-torso cue consisted of the operation of both tactors around the participant’s waist for 150 ms, followed by the operation of both tactors on their hands for 150 ms. Both the cues contained one cycle of “150-150 ms” stimuli, and the total duration of each vibrotactile cue was 300 ms.

Two side loudspeakers positioned to the left and right, 100 cm in front of the participant and approximately 40° to the left and right of the participant’s body midline, were used to present the auditory stimuli. The auditory cues were presented to indicate the direction in which the participant had to turn his or her head for the color discrimination task. They consisted of double tone bursts (15 ms on, 10 ms off, and 15 ms on) presented at 66 dB(A). Pink noise was also delivered at 60 dB(A) from the two side loudspeakers throughout the course of the experimental session in order to mask any noise produced by the operation of the tactors.

The frontal visual stimuli consisted of a moving dot field with gray dots traveling from the center to the edge of the screen against a black background. The dots traveled on a randomized path, and their travel speed varied as a function of the angle of depression of the accelerator, thus providing the participant with a sense that the travel speed of his or her own vehicle varied as a function of the depression angle of the accelerator. A three-dimensional ball presented at the center of the screen served as the “car” in front. The color of the lead object was yellow when the accelerator pedal was depressed more than one third but less than two thirds of its complete depression; otherwise, the lead object turned gray to highlight the fact that the participant was “driving” either too fast or too slow. The lead object had an original radius of 2° ± 0.5° of visual angle, which was randomly chosen prior to the start of each trial.

### Design and Procedure

The participants were seated comfortably and held the steering wheel in front of them with both hands. They were instructed to imagine that they were following a car in front of them (represented by the ball at the center of the screen). They had to try and maintain a safe distance with respect to the lead object by keeping the accelerator at the position between one third and two thirds of its full depression with their right foot such that the color of the lead object stayed yellow. Now and then, a closing-in event would occur, whereby the radius of the lead object would expand rapidly (by 3° in 1,000 ms) in order to simulate a lead vehicle suddenly hitting the brakes. The participants were instructed to brake as soon as they detected the expansion of the lead object, which remained at its expanded size until a participant’s response (i.e., braking) was detected or if no response had been detected within 2 s of the onset of the closing-in event. The lead object then contracted to the next randomized size of 2° ± 0.5° of visual angle within 1 s, and a new trial was started.

**Figure 2.**
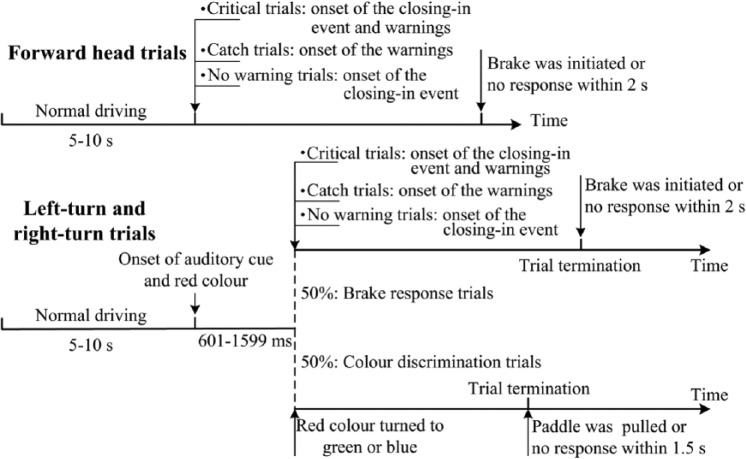
The design and procedure of Experiment 1.

The experimental session consisted of a block of 16 practice trials followed by three blocks of 50 experimental trials. Among the 150 experimental trials, 30 were the forward-head trials, whereas the left turn and right turn contained 60 trials, respectively (see Figure 2). In the former condition, a critical trial (i.e., a closing-in event was started and the warning was presented simultaneously), a catch trial (i.e., the warning was presented without the closing-in event), or a no-warning trial (i.e., a closing-in event was presented without a warning) would be started after a 5- to 10-s duration of driving. The warning signals in the catch trials were counterbalanced between toward-torso and away-from-torso cues. The ratio of critical trials with toward-torso cues to critical trials with away-from-torso cues to catch trials to no-warning trials was 4:4:1:1.

In the left-turn-head and right-turn-head trials, the auditory cue (double tone burst) was respectively presented from the left and right loudspeaker after a 5- to 10-s driving duration. The onset of the red light on the cued side coincided with the presentation of the auditory cue. The participants were instructed to turn their head immediately to the side indicated by the auditory cue and keep looking at the red light. In 50% of these trials (either in the left-turn or in the right-turn trials), the red light turned blue or green after a random interval of 601 to 1,599 ms. The participants had to respond to the color change of the LED by pulling the corresponding hand paddle (the corresponding patterns between the color and the paddles were counterbalanced across participants). The target light remained on until after a response had been made or 1,500 ms had passed since the onset of the color change with no response having been detected. Among the remaining 50% of trials, a critical trial, a catch trial, or a no-warning trial—all of which were designed to be the same as that in the forward-head trails—would be initiated after a random interval of 601 to 1,599 ms. The participant was instructed to return his or her head to the forward position whenever he or she felt the warning signal and determine whether or not there was a closing-in event. If so, the participant had to depress the brake pedal as rapidly as possible. When a participant’s response (i.e., braking) was detected or if no response had been detected within 2 s of the onset of the closing-in event, the lead object then contracted to the next randomized size of 2° ± 0.5° of visual angle within 1 s, and a new trial was started.

There were 12 trials in each combination of head position (forward, turned to left, and turned to right) and vibrotactile warning (toward torso and away from torso). The sequence of all the trials was randomly assigned.

### Results

BRTs, defined as the time after the onset of a critical driving event when the participant initiated a braking response by depressing the brake pedal to over one third of its complete depression, were measured. The BRT data when the participant’s head was turned left and right were combined because an initial repeated-measure analysis of variance (ANOVA) revealed there to be no significant difference between these two conditions, *F*(1, 15) < 1, *p* = .344. Those occasions on which the participants failed to respond before the termination of a trial (i.e., misses) were discarded from the analysis of the response-time data. On average, these misses accounted for less than 1% of all trials in which a warning signal was presented.

**Figure 3.**
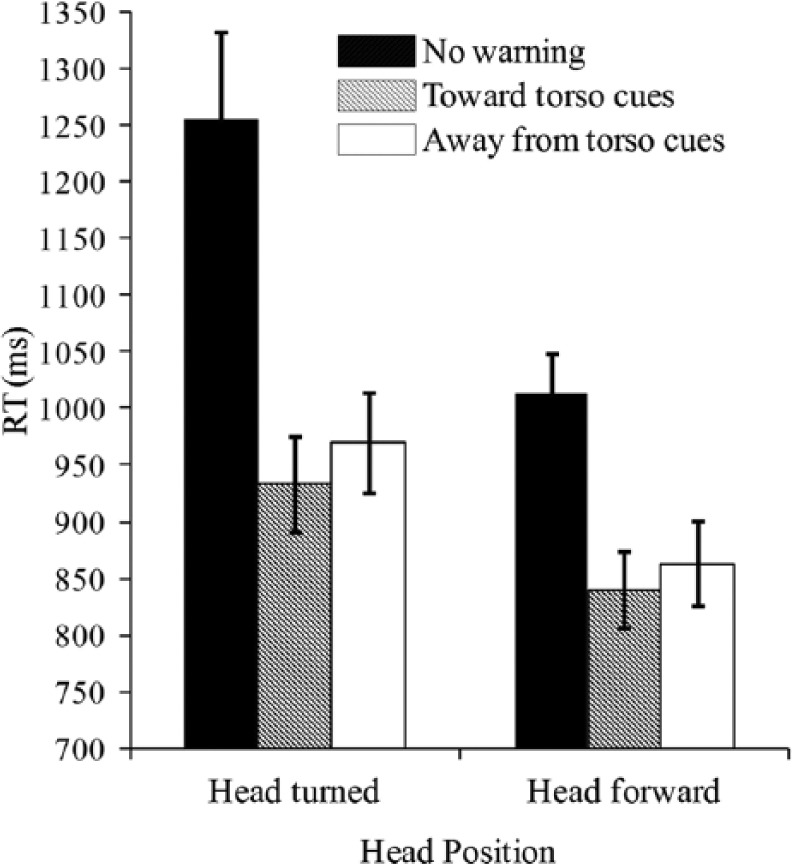
Mean latency of speeded braking responses (RT; in milliseconds) as a function of the warning signal type and head position in Experiment 1. Error bars indicate the standard errors of the means.

A repeated-measure ANOVA was performed on the BRT data in order to determine whether the presentation of the different types of vibrotactile warning signals facilitated participants’ collision avoidance responses. The two factors were warning signal type (no warning, toward-torso cues, away-from-torso cues) and head position (head turned, head forward). As shown in Figure 3, the analysis of the data revealed a significant main effect of warning signal type, *F*(2, 30) = 39.8, *p* < .001. Post hoc comparison with Bonferroni correction revealed that participants responded significantly more rapidly following the presentation of a toward-torso cue (*M* = 886 ms) than when an away-from-torso cue had been presented (*M* = 915 ms, *p* = .016). Overall, on average, the participants missed 3.5% and 0.0% of the trials in which no warning signal had been presented prior to the closing-in event in the head-turned position and head-forward conditions, respectively. Consistent with our expectations, participants responded significantly more slowly when there was no warning signal (*M* = 1,134 ms) than when a warning signal had been presented (both *p* < .001). There was also a significant main effect of head position, *F*(1, 15) = 15.8, *p* = .001. Participants responded significantly more rapidly when looking forward (*M* = 851 ms) than when looking to the left or right (*M* = 951 ms, *p* = .009), as expected.

The average rate of false-alarm braking responses for the two types of warning cues were as follows: toward-torso cue, 28.4% (*SE* = 6.2%); away-from-torso cue, 32.5% (*SE* = 8.6%). A nonparametric test (the Wilcoxon test) revealed that the effect of warning signal type did not reach statistical significance, *Z* = −0.55, *p* = .582.

Performance in the color discrimination task was also measured in order to determine whether the participants had performed the attentional-distraction task as instructed. Those responses occurring 1,500 ms or longer after the presentation of the color cue were discarded as invalid. On average, this elimination led to the removal of 1.8 out of the 60 color discrimination trials (3.0%). The participants correctly responded to the color discrimination targets in valid trials with a mean response time of 769 ms (*SE* = 36 ms) and an accuracy of 93.2% (*SE* = 1.3%), showing that the participants responded to the color discrimination tasks as instructed.

### Discussion

Consistent with the findings of earlier studies, the results of Experiment 1 revealed a significant advantage for vibrotactile cues in facilitating a participant’s braking responses to potential frontal collision in a simulated driving task (e.g., see Chun et al., 2012; Ho, Tan, & Spence, 2005; Ho, Reed, & Spence, 2007; Murata et al., 2011; Scott & Gray, 2008). In addition, Experiment 1 also revealed a significant advantage when the warning signal moved toward the participant’s torso as compared to when it appeared to move away from him or her.

It is important to note, however, that by themselves, the results of Experiment 1 do not demonstrate the effectiveness of the toward-torso vibrotactile warnings in rear-end collision avoidance without some form of comparison with a baseline (static vibrotactile) condition. Additionally, the fidelity of the driving simulation utilized in Experiment 1 was low. Therefore, in order to address the applicability of our results to a more realistic driving situation, in Experiment 2, we replicated Experiment 1 in a medium-fidelity driving simulator and added a static vibrotactile condition.

## Experiment 2

### Participants

Sixteen participants (mean age of 23.4 years, age range from 19 to 27 years; 11 males) took part in this study. All of the participants held a full driving license and had been driving for between 2 and 8 years (mean of 3.9 years). All of the participants reported having normal or corrected-to-normal vision and a normal sense of touch. The experiment lasted for approximately 90 min, and each participant completed an informed-consent form and received course credit in return for participation. The work reported in Experiment 2 was approved by the Science, Technology, Mathematics, and Engineering (STEM) Ethical Review Committee at the University of Birmingham.

### Apparatus

**Figure 4.**
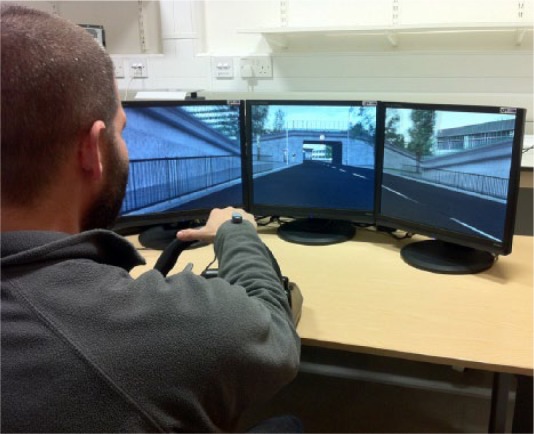
The driving simulator setup in Experiment 2.

An XPI Simulation Limited™ XPDS-XP300 driving simulator (Version 2.2; see Figure 4) was used in a completely dark laboratory setting. The driving simulator comprised a steering wheel (G25 Racing Wheel, Logitech™) with force feedback, a set of pedals, and three Microsoft Plug and Play monitors with 43.2-cm (2,840 × 1,025 pixels resolution) displays. External PC loudspeakers, positioned adjacent to the monitors, were used to present auditory cues. The participant was seated comfortably in front of the steering wheel, with an approximately 80-cm view distance to the monitors. The gas and brake pedals were also placed at a comfortable distance from the participant.

The vibrotactile stimuli were presented via two pairs of vibrational tactors (VBW32, Audiological Engineering Corp., Somerville, MA, USA). All of the tactors were driven by a 250 Hz sinusoidal signal at an intensity that was sufficient to deliver perceivable vibrotactile stimuli. One pair of tactors was fastened to the back of the participant’s hands and the other pair to the left and right sides of his or her waist using Velcro belts. Three types of vibrotactile signals were tested in this experiment: The dynamic toward-torso cues consisted of the simultaneous operation of both tactors on the participant’s hands for 150 ms, immediately followed by the simultaneous operation of the two tactors on his or her waist for a further 150 ms; the dynamic away-from-torso cue consisted of the simultaneous operation of both tactors on the participant’s waist for 150 ms, immediately followed by the simultaneous operation of the two tactors on his or her hands for a further 150 ms; and the static cues consisted of the simultaneous operation of the two pairs of tactors on the participant’s hands and waist for 300 ms. All warnings had a total duration of 300 ms. The overall intensity level across the three types of warnings was matched.

### Design and Procedure

The participants were instructed to follow a lead vehicle in a rural, two-way road with traffic passing in the other lane in the opposite direction. They were required to drive in their own lane and to try and maintain a 2.0-s time headway (TH) with the lead vehicle. If the participant followed too far behind the lead vehicle, the phrase “Speed up” was presented from the loudspeaker. There was no “slow down” warning, so the participant was free to maintain any TH lower than 2 s. The participants were given a 5-min practice drive without any warning signals in order to familiarize themselves with the driving simulator. Each participant completed four blocks of 20 trials corresponding to the three types of vibrotactile warning signals plus a no-warning condition. The order of the test blocks was counterbalanced and was selected at random. Each block required roughly 20 min, and participants took a 5-min rest between blocks to minimize the simulator sickness and fatigue.

At the start of the critical trial, the lead vehicle accelerated to 60 mph from a stationary position and then was programmed to unpredictably (to the driver) change speeds between 55 mph and 65 mph (with an average speed of 60 mph), changing speed on average once every 5 s. At a random time interval (between 30 and 90 s after the start of the trial), the lead vehicle suddenly braked at a rate of −6 m/s^2^ to come to a full stop. The behavior of the lead car made it very difficult for the driver to predict when the lead car would speed up, slow down, or stop, thus creating multiple possible rear-end collision situations. In order to reduce the frequency of the warning presentation as compared to Experiment 1, each block consisted of 16 randomized critical trials (i.e., the lead vehicle made a sudden brake) and 4 noncritical trials (i.e., no sudden brake for the lead vehicle). In the critical trials of the three blocks with warnings, the vibrotactile signal was presented simultaneously with the deceleration of the lead vehicle. The participants were instructed to avoid any collisions with the lead vehicle by braking and were instructed not to depart from the lane. Trials with lane departures were discarded and repeated. The critical trial was programmed to end when the participant’s vehicle came to a complete stop or when it collided with the lead vehicle, and the noncritical trial would be ended after a time interval of 60 s. A new trial was then started.

### Results

A one-way repeated-measures ANOVA with the warning condition (no warning, toward-torso cues, away-from-torso cues, and static cues) as the sole factor was performed in order to determine whether the presentation of different warnings facilitated the drivers’ BRTs.

**Figure 5.**
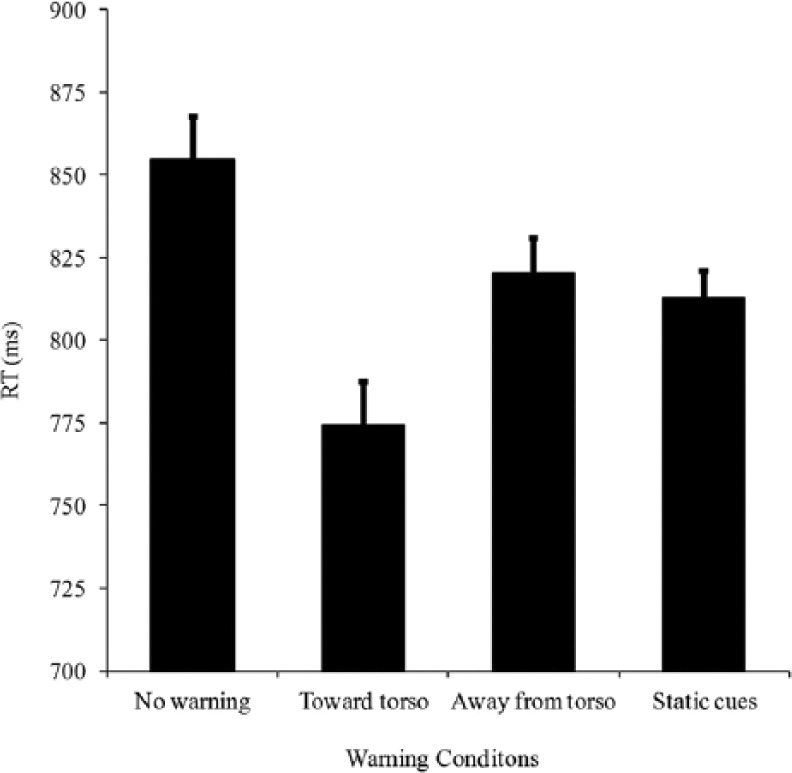
Mean latency of speeded braking reaction times (in milliseconds) as a function of the warning signal type in Experiment 2. The error bars indicate the standard errors of the means.

As shown in Figure 5, the ANOVA results revealed a significant main effect of the type of warning signal, *F*(3, 45) = 9.6, *p* < .001. Post hoc pairwise comparisons with Bonferroni correction revealed that the participants responded significantly more rapidly following the toward-torso cues (*M* = 766 ms) than following the other types of warning signals (away-from-torso cues, *M* = 820 ms, *p* = .014; static cues, *M* = 813 ms, *p* = .008; no-warning condition, *M* = 855 ms, *p* < .001). The difference between participants’ BRTs following the presentation of away-from-torso cues and the no-warning condition only approached significance (*p* = .081). No significant difference was revealed between the away-from-torso cues and the static cues (*p* = .577).

### Discussion

Crucially, the findings of Experiment 2 replicated those documented in Experiment 1. In particular, the toward-torso warning signals were once again demonstrated to be more effective than the away-from-torso warnings in terms of speeding participants’ BRTs. Moreover, the results of Experiment 2 further demonstrated the advantage of toward-torso warnings over the static vibrotactile warnings. These results can therefore be taken to suggest that the toward-torso vibrotactile warning signal was informative in terms of preparing the participant/driver to perceive a frontal closing-in event coming in the direction identical to that of the vibrotactile warning signal. It was also likely that the vibrotactile warning signals that appeared to travel toward the participant’s torso might somehow have tapped specific “threat-related” circuitry in the participant’s brain that would presumably have enhanced the early stages of visual information processing (see Leo, Romei, Freeman, Ladavas, & Driver, 2011) and may have triggered the participants’ defensive reaction to speed braking responses (see Freiberg, Tually, & Crassini, 2001; Ho & Spence, 2009; Yonas et al., 1977).

Based on this explanation, it could be imagined that should the cues associated with the perceived approach toward the torso be strengthened, the effect of toward-torso cues on the speeding of a driver’s reactions to time-critical events might be even stronger. In the field of research on auditory warnings, increasing the intensity of a warning sound can produce an impression of a sound-emitting object approaching the observer (see Shaw et al., 1991). It therefore seemed possible that increasing the intensity of a vibrotactile warning signal could also be used to strengthen the perceived approach toward the driver. Previously, it has been demonstrated that change in the intensity of vibrotactile cues could be used to convey information about the proximity of a vehicle to an obstacle in a driving environment (see Jones & Sarter, 2008). Therefore, in our third experiment, we attempted to strengthen the perceived approach of the vibrotactile stimuli toward the participant’s torso by introducing some variation in the intensity of the tactile signal. In Experiment 3, two types of vibrotactile toward-torso warning signals were examined: the first with low vibration intensity on the hand tactors and high-intensity vibration on the waist tactors, and the second with an identical vibration intensity on both the hands and waist. Our hypothesis was that the cues with increasing vibration intensity from hands to waist would produce a stronger sense of approach toward the body than those cues having a constant vibration intensity on both the hands and waist and that this effect would result in a more effective warning signal in terms of facilitating a driver’s responses to potential closing-in events.

## Experiment 3

### Method

Sixteen participants (mean age of 27 years, age range from 18 to 40 years; 9 males) took part in this experiment. Seven of the participants reported that they would normally drive on the left, and the remainder reported that they would normally drive on the right side of the road. All of the participants reported having normal or corrected-to-normal vision, normal hearing, a normal sense of touch, and no history of neck pain. Five of the participants had previously taken part in Experiment 1.

The apparatus, materials, design, and procedure were all identical to those used in Experiment 1, with the sole exception that the warning signal type now consisted of two toward-torso vibrotactile cues, one with low cue intensity on hands and high cue intensity on waist (looming-intensity toward-torso cue), and the other with identical cue intensity on the hands and waist (constant-intensity toward-torso cue). The looming-intensity toward-torso cue involved the presentation of weak vibrotactile stimuli (approximately one third of the physical intensity of the stimuli utilized in Experiment 1) on both hands and then presented vibrotactile stimuli at a strong intensity (the same as the intensity in Experiment 1) on the waist. The constant-intensity toward-torso cue involved the presentation of vibrotactile stimuli at a high intensity (the intensity was the same as in Experiment 1) on both the hands and waist. All of the vibrotactile stimuli were presented at an intensity that was clearly perceived by our participants. The two types of toward-torso cues consisted of the simultaneous operation of both tactors on the participant’s hands for 150 ms, followed consecutively by the simultaneous operation of the two tactors on the waist for 150 ms. The experiment lasted for 30 min. The participants all signed an informed-consent form, and each received £5 in return for taking part.

### Results

As in Experiment 1, an ANOVA was performed on the BRT with the within-participants factors of warning signal type (no warning, looming-intensity toward-torso cue, constant-intensity toward-torso cue) and head position (head turned, head forward). Those occasions in which the participants failed to respond before the termination of a trial (i.e., misses) were discarded from the analysis of the BRT data. On average, the participants missed less than 1% of all trials in which a warning signal was presented.

**Figure 6.**
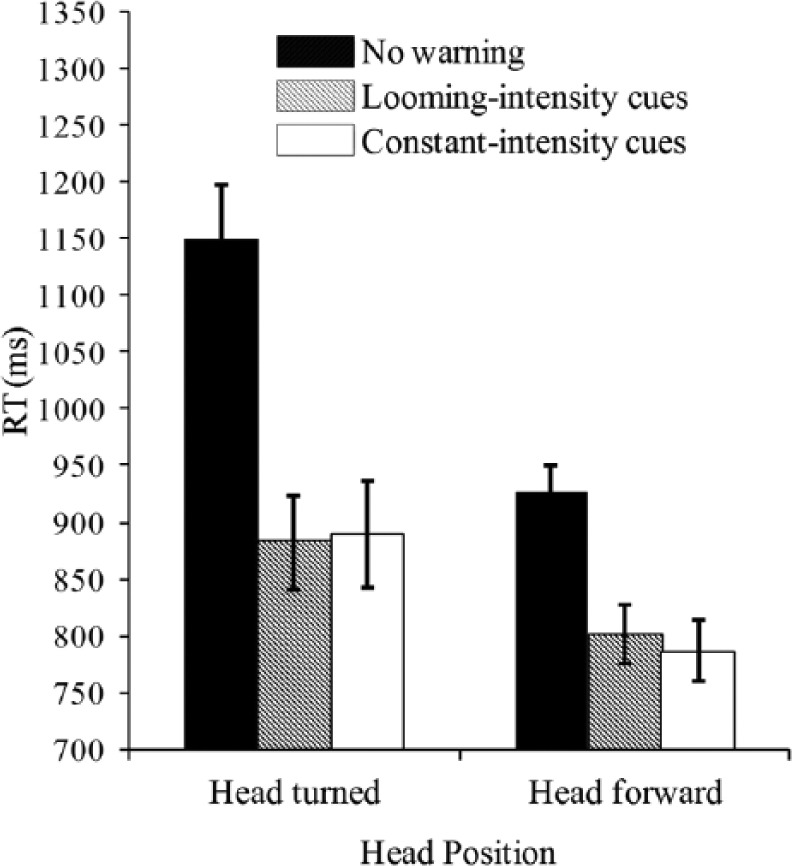
Mean latency of speeded braking responses (RT; in milliseconds) as a function of the warning signal type and head position in Experiment 3. Error bars indicate the standard errors of the means.

Overall, the participants missed an average of 2.0% and 0.0% of the trials in which no warning signal was presented prior to the closing-in event in the head turned and head forward conditions, respectively. As shown in Figure 6, the analysis revealed a significant main effect of warning signal type, *F*(2, 30) = 30.9, *p* < .001. Post hoc comparison with Bonferroni correction revealed that participants responded significantly more slowly when there was no warning signal (*M* = 1,075 ms) than when there was either a looming-intensity cue (*M* = 842 ms, *p* < .001) or a constant-intensity cue (*M* = 838 ms, *p* < .001). However, there was no significance between the looming-intensity and constant-intensity cues, *ns*. The data analysis also revealed a significant main effect of head position, *F*(1, 15) = 12.5, *p* = .003. The participants responded significantly more rapidly when they were looking forward (*M* = 794 ms) than when they were looking to the left or right (*M* = 886 ms) at the onset of the warning signals.

The average rate of false-alarm braking for the two types of warning cues were as follows: looming-intensity toward-torso cue, 26.6% (*SE* = 7.5%); constant-intensity toward-torso cue, 25.3% (*SE* = 8.5%). A nonparametric test (the Wilcoxon test) revealed that there was no effect of warning signal type, *Z* = −0.408, *p* = .683.

Performance in the color discrimination task was analyzed in the same manner as in Experiment 1. On average, 3.8 out of the 60 color discrimination trials (6.3%) were discarded. The participants correctly responded to 90.2% (*SE* = 2.1%) of the color discrimination targets in valid trials (i.e., the trials in which a response was detected within 1,500 ms of the presentation of the color cue), with a mean response time of 814 ms (*SE* = 43 ms). Their performance in this task was similar to that observed in Experiment 1.

### Discussion

Our hypothesis was that the presentation of looming-intensity vibrotactile cues that moved from the participant’s hands to his or her waist would strengthen the sense of motion toward his or her torso and thus potentially offer additional benefit in terms of facilitating the driver’s braking responses than the constant-intensity vibrotactile cues. However, contrary to our expectations, the results of Experiment 3 failed to demonstrate any significant difference between the two types of vibrotactile cue. It would therefore seem as though the looming intensity of the vibrotactile cue did not provide any further useful information about the collision events that the participants could use to help speed their responses.

Based on the results of the three experiments reported so far, it is unclear whether our results can be taken to suggest that a cue that appeared to move toward the participant’s torso connoted the motion of an external event approaching the driver. The toward-torso cue presented vibrotactile stimuli on the participant’s hands first (150 ms earlier), whereas an away-from-torso cue presented vibrotactile stimuli to the waist first. The benefit of the toward-torso cue in facilitating a driver’s braking responses might therefore have resulted from the fact that the drivers responded to potential collision events more rapidly when the stimuli were presented on their hands than when presented on their waist (though see Ho & Spence, in press, on this point) but not from the perceived motion direction (if any) of the toward-torso cue. In addition, the results of Experiment 3 were ambiguous with respect to the effectiveness of the looming-intensity cue in terms of providing additional benefits in a driver’s collision avoidance responses, since the looming-intensity cue had a lower overall intensity than the constant-intensity signal. The difference in the intensity of the vibration between these two types of warning signals might have been expected to result in different effects on a driver’s braking responses. Therefore, it may possibly have been the case that any benefit offered by the looming-intensity component of the looming-intensity cues was balanced out by that offered by the higher overall vibration intensity in the constant-intensity vibrotactile cue case, resulting in no significant difference in the observed responses.

In order to further understand the effect of toward-torso motion and looming-intensity vibrotactile cues, we conducted a fourth and final experiment in which the vibrotactile cues were presented only on the participant’s hands or waist, with either low or high vibration intensity. The final experiment was designed to investigate (a) whether there was some difference in the effect of vibrotactile cues presented on hands and on the waist and (b) whether the intensity of the vibrotactile cues would exert a significant effect on a driver’s braking responses, particularly when the vibrotactile stimuli were presented on his or her hands.

## Experiment 4

### Method

Twenty participants took part in this experiment. The data from two participants were excluded because of equipment failure. As a result, the data from 18 of the participants (10 male; mean age of 27 years, age range 18 to 40 years) were included in the following analyses. All of the participants had a valid U.K. or international driving license and had, on average, been driving for 7 years (ranging from 1 to 23 years). Seven of the participants reported that they would normally drive on the left, and the remainder reported that they would normally drive on the right. All of the participants had normal or corrected-to-normal vision, normal hearing, a normal sense of touch, and no history of neck pain. Seven of these participants had taken part in Experiments 1 or 3.

The apparatus, materials, design, and procedure were identical to those used in Experiment 3, with the exception that the four types of warning signal were included as cues: a low cue intensity to both hands (one third of the physical intensity in Experiment 1), a high cue intensity on both hands (the same as intensity in Experiment 1), a low cue intensity on the waist (one third of the physical intensity used in Experiment 1), and a high cue intensity on the waist (the same intensity as in Experiment 1). All four types of vibrotactile cue consisted of the simultaneous operation of both tactors on the participant’s hands/waist for 150 ms. The experimental session consisted of a block of 16 practice trials followed by three blocks of 100 experimental trials. The participants were given a short break between blocks. Among the 300 experimental trials, 60 trials were the forward-head trials, and the left-turn and right-turn trials consisted of 120 trials, respectively. The ratio of critical trials to catch trials to no-warning trials was 8:1:1. The proportion of the four types of warning signal in the critical trials was equal. There were 12 trials in each combination of head position (forward, turned to left, and turned to right) and vibrotactile warnings (low intensity on hands, high intensity on hands, low intensity on waist, and high intensity on waist). The sequence of trials was randomly ordered. The experiment lasted for about 60 min in total, and the participants signed an informed-consent form and received £10.

### Results

Similar-analyses to those performed in Experiments 1 and 3 were performed on the BRT data from Experiment 4. A repeated measures ANOVA with the within-participants factors of warning location (on hands vs. on waist), cue intensity (low vs. high), and head position (head turned vs. head forward) was performed in order to compare the relative effectiveness of the four types of vibrotactile warning signal in terms of orienting participants’ attention in preparing to make a speeded braking response. Those occasions when the participants failed to respond before the termination of a trial (i.e., misses) were discarded from the analysis of the response-time data. On average, the participants missed less than 1% of all trials in which a warning signal had been presented.

**Figure 7.**
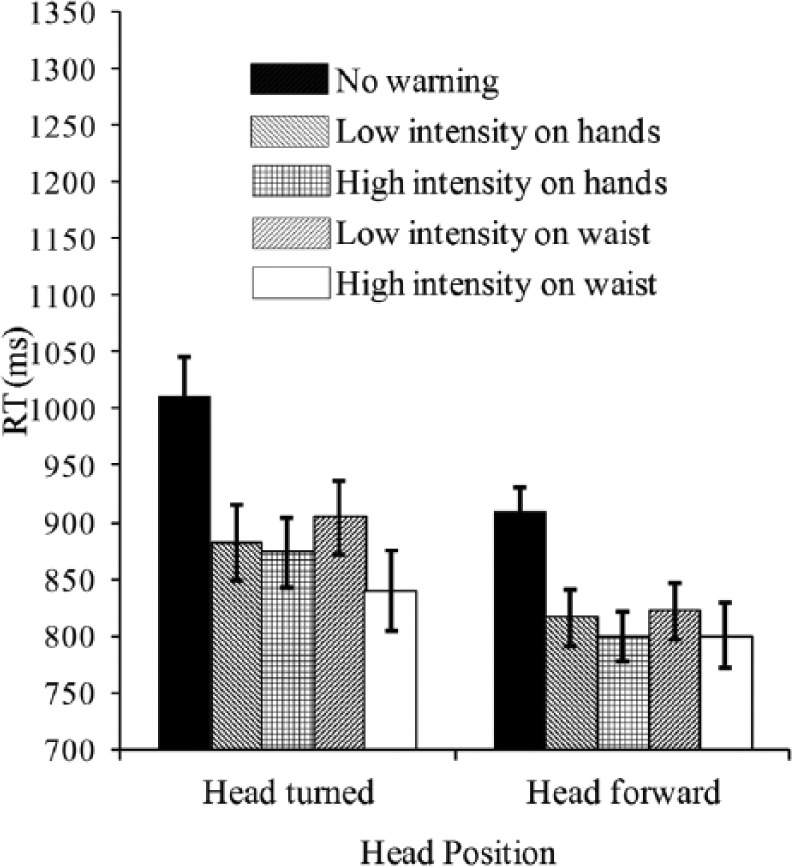
Mean latency of speeded braking responses (RT; in milliseconds) as a function of the type of warning signal and head position in Experiment 4. Error bars indicate the standard errors of the means.

The analysis of the BRT data revealed no main effect of warning location (on the hands, *M* = 843 ms; on the waist, *M* = 841 ms), *F*(1, 17) < 1.0, *p* = .907 (see Figure 7). The analysis revealed a significant main effect of cue intensity (low intensity, *M* = 856 ms; high intensity, *M* = 828 ms), with *F*(1, 17) = 24.2, *p* < .001. Further comparison indicated that when the cues were presented on the participant’s hands, the difference between the low- and high-intensity cues (*M* = 849 ms and 836 ms, respectively) was not significantly different, *F*(1, 17) = 1.7, *p* = .211. When the cues were presented on the participant’s waist, the responses were more rapid following the presentation of the high-intensity cues than following the presentation of the low-intensity cues (high intensity, *M* = 820 ms; low intensity, *M* = 863 ms), *F*(1, 17) = 14.7, *p* = .001. Once again, the analysis revealed a significant main effect of head position, *F*(1, 17) = 10.5, *p* = .005. Participants responded significantly more rapidly when they were looking forward (*M* = 809 ms) than when they were looking to the side (*M* = 875 ms) at the onset of the warning signal.

On average, the participants missed 2.8% and 0.5% of the trials in which no warning signal was presented prior to the closing-in event in the head-turned conditions and head-forward conditions, respectively. Pairwise comparison (repeated-measures ANOVA) on the response-time data between the warning signal absent and present conditions revealed that the participants responded significantly more slowly in the absence of a warning signal (*M* = 977 ms) than when a warning signal was presented: no warning versus low intensity on hands, *F*(1, 17) = 22.2, *p* < .001; no warning versus high intensity on hands, *F*(1, 17) = 30.1, *p* < .001; no warning versus low intensity on the waist, *F*(1, 17) = 22.4, *p* < .001; no warning versus high intensity on waist, *F*(1, 17) = 27.9, *p* < .001, as expected.

The average rates of false-alarm braking for the four types of warning cues were as follows: for a vibrotactile cue with low intensity delivered to the hands, 4.2% (*SE* = 2.2%); for a vibrotactile cue with high intensity on the hands, 22.3% (*SE* = 4.3%); for a vibrotactile cue with low intensity delivered to the waist, 7.5% (*SE* = 3.0%); and for a vibrotactile cue with a high intensity on the waist, 10.9% (*SE* = 5.1%). The results of a nonparametric test (Friedman’s test) revealed a significant effect of warning signal on the rate of false alarm braking, χ^2^(3) = 19.5, *p* < .001. Post hoc pairwise comparison revealed that when the vibrotactile cues were presented on the driver’s hands, the participants initiated more false-alarm braking following the high-vibration-intensity cue than that following the low-vibration-intensity cue (*p* = .002). The rate of false-alarm braking was also significantly higher following the presentation of high-intensity vibration on the participant’s hands than following high-intensity vibration presented to the waist (marginally, with *p* = .054). None of the other comparisons were significant, all *p* > .416.

Performance in the color discrimination task was analyzed in the same manner as for Experiments 1 and 3. Those responses occurring 1,500 ms or more after the presentation of the color cue were discarded as invalid. On average, this elimination resulted in 7.2 out of 120 color discrimination trials (6.0%) being removed. The mean percentage of correct responses in the color discrimination task in valid trials was 89.4% (*SE* = 2.3%), with a mean response time of 917 ms (*SE* = 33 ms). Performance in this task was similar to that observed in Experiments 1 and 3.

### Discussion

The results of Experiment 4 revealed no significant difference between the cues presented on the participants’ hands and those presented on their waist, when the intensity of the stimuli was either low or high. These results therefore suggest that the advantage of toward-torso over away-from-torso cues reported in Experiment 1 cannot simply be attributed to drivers’ responding more rapidly to the vibrotactile warnings presented on their hands than to those presented on their waist. Instead, these results may reflect the fact that the perceived direction of the toward-torso dynamic vibrotactile cue was compatible with the direction of motion of the external object, which provided some information concerning the frontal collision events that consequently facilitated a driver’s response to the potential collision.

## General Discussion

Authors of previous research have investigated the effectiveness of dynamic upward and downward vibrotactile moving cues presented by the sequential activation of three tactors aligned along the driver’s midline (Ho et al., 2014). The results of this research suggested that the direction of travel dynamic vibrotactile cues failed to give rise to any significant difference in terms of facilitating the driver’s time-critical responses to the critical driving events. However, the BRT data following the presentation of the dynamic toward-torso and away-from-torso vibrotactile cues in the present study (see Experiments 1 and 2) highlighted a significant advantage of the toward-torso cues over the away-from-torso and static vibrotactile cues. It should, however, be noted that although the vibrotactile cues in both studies were presented in the driver’s peripersonal space, they had a distinctive motion direction relative to that of the driver’s body. The vibrotactile cues in Ho et al.’s (2014) study (upward and downward on the torso) provided a type of vertical apparent motion going toward/away from the head; in contrast, the vibrotactile cue in the present study (toward and away from the torso) likely gave rise to lateral apparent motion toward/away from the torso.

At this point, at least three possible explanations of the superiority of toward-torso over other types of vibrotactile cues should be considered. First, the toward-torso cues presented stimuli on the hands first, and the advantage of this type of cue might therefore have resulted from the fact that the drivers responded to potential collisions more rapidly when the stimuli were presented on their hands than when presented on their waist. However, the results of Experiment 4 highlighted the absence of any significant difference between the vibrotactile cues presented on the participants’ hands and on their waist. These results therefore demonstrate that the first account does not provide a particularly satisfactory explanation for the superiority of the toward-torso cues documented in the present study.

Second, the performance facilitation seen following the dynamic toward-torso vibrotactile warnings may have occurred because the perceived moving direction of the warning (approaching the participant from hands to waist) is consistent with the direction of motion in the potential rear-end collisions. Such directional mapping might then help to convey more information concerning the potential frontal collision to the participants and thus facilitate their visual information processing (see Leo et al., 2011).

Third, the dynamic toward-torso vibrotactile warnings might have a higher urgency associated with them and could thus serve to better disengage a participant’s attention from whatever task he or she is otherwise engaged in, no matter the direction of the external events. Such attentional facilitation leads to participants’ responding more rapidly and exhibiting improved behavioral performance. Previous studies have demonstrated that in the auditory modality, when a sound moves toward an observer, it has greater attentional salience than the same sound when it is perceived to be moving away (e.g., Hall & Moore, 2003; see also Leo et al., 2011). Similarly, the toward-torso cue might potentially be expected to trigger a “danger-approaching” signal in the brain, thus inducing a higher level of urgency and thus give rise to an alerting effect, which may encourage an organism (in this case, the driver) to avoid the danger. Given that the away-from-torso cue can be interpreted as something moving away from the body, this cue may have resulted in a low perceived urgency of the warning signal, thus slowing the participants’ braking responses.

The results of Experiment 4 failed to reveal any significant effect of cue intensity on a driver’s response to potential collision events when the cue was presented on his or her hands. Combining this result with those of Experiment 3, it would appear that the looming intensity (with weak intensity on hands and strong intensity on waist) in the toward-torso cue did not provide any advantage over the constant-intensity cues in terms of facilitating a driver’s collision avoidance responses. Previous research has revealed that giving the intensity increase a looming profile on a single spot on the skin surface did not stand out from other nonlooming vibrotactile cues (see Gray et al., 2014; Ho et al., 2013). Our results further reveal that the effect of looming intensity in the context of a directional vibrotactile cue was limited. When taken together, the results of the present study would appear to suggest that intensity does not necessarily provide an effective means of conveying information in the tactile modality (in contrast to the advantage that has been observed in audition; see Gray, 2011).

Although our participants responded significantly more rapidly following the toward-torso warnings than following the away-from-torso warnings, the difference in BRTs between the two types of warning signals was relatively small (approximately 30 ms in Experiment 1 and 50 ms in Experiment 2). It should, however, be noted that the relatively small effect may well have resulted from the repetitive and simple responses required of participants in the present study. After the presentation of the vibrotactile stimuli, the only task for the participant was to check whether the lead object was approaching and determine whether to brake or not. The simple task required relatively little time to complete, no matter the type of the warning signal. However, that said, it is worth bearing in mind that a 30-ms reduction in braking latencies equates to stopping the car approximately 1.0 m earlier at the speed of 120 km/h (similarly, a 50-ms reduction in BRTs means approximately 1.7-m shorter stopping distance for a car travelling at 120 km/h). We would argue that such effects, albeit small, could nevertheless contribute in some small way to reducing the occurrence and/or the severity of the rear-end collisions on roads.

The rate of false-alarm braking was relatively high in Experiments 1, 3, and 4. This high false-alarm braking rate might be due to the high reliability of the warning signal and the repetition of the simple task reaction. First, previous research suggests that drivers (and other interface operators) may ignore or disable collision-warning systems if the warnings do not reliably signal potential danger (Bliss & Acton, 2003; Gray, 2011). Therefore, the reliability of the warning signal tested in the present experiments was set at a high value (approximately 90%), and high warning reliability is usually associated with high false-alarm reactions (see Bliss & Acton, 2003; Graham, 1999). Second, the participants’ task in the present experiments was relatively simple (they needed only to hit the brake pedal whenever they detected a closing-in event) and highly repetitive. In such tasks, some kind of “reflex action” between the warning signal with high reliability and the braking responses might be triggered after being repeated many times, thus possibly resulting in a relatively high false-alarm braking rate. Research conducted in a driving simulator has revealed there to be no significant difference between participants’ steering response times when the rate of false-alarm response was low or high (Bliss & Acton, 2003). Therefore, the high rate of false-alarm braking might not produce much influence on the comparison of participants’ BRTs as a function of the presentation of different vibrotactile cues.

A number of limitations associated with the present study should be acknowledged. First, the experimental settings and the simulated tasks in the present experiments (except for Experiment 2) had a low ecological validity to the realistic driving environment. Second, in order to investigate participants’ average BRTs under different vibrotactile warnings within a reasonable time frame, in the present study (Experiments 1, 3, and 4), we adopted the paradigm with a highly repetitive trial structure. It should be remembered that drivers in actual driving situations would presumably mostly find potential rear-end collisions to be a very rare occurrence in real life (Parasuraman, Hancock, & Olofinboba, 1997; Spence & Ho, 2008b), and they might not respond to such infrequent vibrotactile warnings as rapidly as in the laboratory experiments (Parasuraman et al., 1997). Although we tried to adopt a paradigm with events occurring somewhat less frequently (approximately 60 trials per hour) in Experiment 2, the presentation frequency of vibrotactile warnings was still much higher than a driver would ever encounter in a realistic driving situation. It will therefore be crucial in future research to investigate the effectiveness of the toward-torso vibrotactile warnings in the condition with rare critical events. Given the limitations previously highlighted, the interpretation of our results should therefore be taken with some degree of caution.

In the application of toward-torso vibrotactile warnings, vibration on the hands and waist could be presented from the steering wheel and the seat belts, respectively (see Beruscha et al., 2011; Chun et al., 2012; Ho et al., 2006, 2007). This design would allow one to generate a toward-torso cue. However, it is worth noting that how drivers hold the steering wheel and where the seat belt happens to fall across their body in real-world driving may impact the effectiveness of the toward-torso vibrotactile warnings. For example, drivers sometimes control the vehicle with only one hand, especially drivers in manual cars. Moreover, the seat belt may not be properly fastened, and drivers may not feel the vibration delivered from the actuators embedded in the belts.

On the other hand, people’s beliefs about the effectiveness of the preventive behavior and the risk of being injured have been revealed to be strongly related to people’s self-protection actions (Lehto & James, 1997). For example, a self-report survey about seat belt usage in Spain (see Cunill, Gras, Planes, Oliveras, & Sullman, 2004) found that more than 95% of the participants reported that they always or almost always used a seat belt on highways, a figure that was significantly higher than that on urban roads (about 60%). Other research also showed similar results (e.g., Eby, Molnar, & Olk, 2000). Further exploration revealed that people considered that the injury risk was much higher and the seat belt would be more effective in preventing injuries on highways than on urban roads. Therefore, people tend to fasten their seat belts more when driving on the highway. Thus, it would seem reasonable to suggest that if drivers perceived the effectiveness of the collision-warning systems in reducing the risk of rear-end collisions, they might just tend to fasten the seat belt at the appropriate position and hold the steering wheel with both hands in order to feel the vibrotactile warnings in a timely manner. Additionally, it has also been reported that drivers tend to use both hands to control their vehicle as the objective risk of driving increases (see Walton & Thomas, 2005). However, there is still much challenge in the implementation of such vibrotactile warning systems. The influence of thick clothes, the vehicle vibration on the roadway, and other practical constraints deserve further investigation.

## Conclusions

Taken together, the results of the four experiments reported in the present study demonstrate the potential benefits of using dynamic tactile cues that approach the torso in order to present time-critical information to drivers. As compared with vibrotactile warnings that travel away from the participant’s torso and those without apparent motion (static cues), the toward-torso vibrotactile warnings offer a particularly effective means of alerting drivers and orienting their attention to the need for subsequent collision avoidance actions. On the other hand, the results of the present study further revealed that embedding looming intensity in the toward-torso vibrotactile warning signal fails to result in any benefit in terms of facilitating a driver’s responses in time-critical driving situations.

In the present study, we did not take the urgency of the closing-in events into consideration. It would therefore be interesting in future research to investigate whether the advantage of the looming intensity in toward-torso warning signals could be detected when the looming rate is made dependent on the closing velocity of the leading car (i.e., urgency of the potential collision; for example, see Gray et al., 2014). In the present study, the effectiveness of vibrotactile warnings in a single braking task was examined. It would also be interesting in future research to investigate the effectiveness of vibrotactile warning signals when other warnings are included, such as lane departure warnings (see Navarro, Mars, & Hoc, 2007) or pedestrian collision warnings (see Straughn, Gray, & Tan, 2009).

## Key Points

- Participants react significantly more rapidly following dynamic vibrotactile cues that appear to move toward the torso than following those cues that move away from the torso and following nonlooming cues.
- Making the intensity of the toward-torso cue loom does not provide any advantage over the toward-torso cue with constant intensity.
- By embedding the approach information concerning a potential frontal collision in the design of the warning signal, dynamic vibrotactile cues can potentially offer benefits that may facilitate a driver’s responses in time-critical driving situations.
